# Effect of Co-segregating Markers on High-Density Genetic Maps and Prediction of Map Expansion Using Machine Learning Algorithms

**DOI:** 10.3389/fpls.2017.01434

**Published:** 2017-08-23

**Authors:** Amidou N’Diaye, Jemanesh K. Haile, D. Brian Fowler, Karim Ammar, Curtis J. Pozniak

**Affiliations:** ^1^Department of Plant Sciences, Crop Development Centre, University of Saskatchewan, Saskatoon SK, Canada; ^2^International Maize and Wheat Improvement Center (CIMMYT) Texcoco, Mexico

**Keywords:** genetic map, high-density, single nucleotide polymorphism, wheat, prediction, map expansion, inflation factor, machine learning

## Abstract

Advances in sequencing and genotyping methods have enable cost-effective production of high throughput single nucleotide polymorphism (SNP) markers, making them the choice for linkage mapping. As a result, many laboratories have developed high-throughput SNP assays and built high-density genetic maps. However, the number of markers may, by orders of magnitude, exceed the resolution of recombination for a given population size so that only a minority of markers can accurately be ordered. Another issue attached to the so-called ‘large p, small n’ problem is that high-density genetic maps inevitably result in many markers clustering at the same position (co-segregating markers). While there are a number of related papers, none have addressed the impact of co-segregating markers on genetic maps. In the present study, we investigated the effects of co-segregating markers on high-density genetic map length and marker order using empirical data from two populations of wheat, Mohawk × Cocorit (durum wheat) and Norstar × Cappelle Desprez (bread wheat). The maps of both populations consisted of 85% co-segregating markers. Our study clearly showed that excess of co-segregating markers can lead to map expansion, but has little effect on markers order. To estimate the inflation factor (IF), we generated a total of 24,473 linkage maps (8,203 maps for Mohawk × Cocorit and 16,270 maps for Norstar × Cappelle Desprez). Using seven machine learning algorithms, we were able to predict with an accuracy of 0.7 the map expansion due to the proportion of co-segregating markers. For example in Mohawk × Cocorit, with 10 and 80% co-segregating markers the length of the map inflated by 4.5 and 16.6%, respectively. Similarly, the map of Norstar × Cappelle Desprez expanded by 3.8 and 11.7% with 10 and 80% co-segregating markers. With the increasing number of markers on SNP-chips, the proportion of co-segregating markers in high-density maps will continue to increase making map expansion unavoidable. Therefore, we suggest developers improve linkage mapping algorithms for efficient analysis of high-throughput data. This study outlines a practical strategy to estimate the IF due to the proportion of co-segregating markers and outlines a method to scale the length of the map accordingly.

## Introduction

Genetic maps also known as linkage maps are constructed for several purposes (see Semagn et al., 2006 for a review). In particular, genetic maps:

– Allow identifying genomic regions that control the expression of qualitative and quantitative trait loci (QTL) (Mohan et al., 1997; Doerge, 2002; Yim et al., 2002).
– Help in marker-assisted selection by facilitating the introgression of desirable QTL.
– Allow phylogenetic analyses between different species for evaluating similarity between genes (Ahn and Tanksley, 1993; Paterson et al., 2000).
– Help in the identification of chromosomal rearrangements (Tanksley et al., 1992; Agresti et al., 2000; Bansal et al., 2007).
– Help in anchoring physical maps (Yim et al., 2002).
– Facilitate *de novo* genome assembly and validation by enabling the identification of chimeric scaffold constructs (Rastas et al., 2013; Fierst, 2015).
– Where high-density maps are required, constitute the first step toward positional or map-based cloning of genes responsible for economically important traits, (Mohan et al., 1997; Vuylsteke et al., 1999).

Genetic maps indicate the position and relative genetic distances between markers along chromosomes, which is analogous to signs or landmarks along a highway where the genes are “houses” (Paterson, 1996; Collard et al., 2005). Genetic maps are constructed using different types and sizes of mapping populations, laboratory techniques, marker systems, mapping strategies, statistical procedures and computer packages. These factors can affect the efficiency of the mapping process (Liu, 1998; Paterson et al., 2000). Map length and marker orders are impacted by various factors, including the type and size of the population (Ferreira et al., 2006), the type of markers (dominant or codominant), genotyping or scoring errors, distortion segregation (Hackett and Broadfoot, 2003; Oliveira et al., 2004) and the frequency of double recombinants.

Advances in sequencing and genotyping technologies have enabled the massive production of single nucleotide polymorphism (SNP) markers in a cost-effective way, making SNP markers the choice for linkage mapping. As a result, many laboratories have developed high-throughput SNP assays with continuously increasing marker numbers. For wheat, there are the 9K (Cavanagh et al., 2013), the 90K (Wang et al., 2014) and the 820K (Winfield et al., 2016) SNP assays. Similar efforts have been made for other crops, including rice with the RICE6K (Yu et al., 2014) and the RiceSNP50 (Chen H. et al., 2014), and maize with the MaizeSNP50 (Ganal et al., 2011) and the 600K (Unterseer et al., 2014).

Indeed, a high number of markers are needed to build high-density genetic maps that are suitable for positional or map-based cloning of genes. However, the disproportion between the high number of markers and the relatively small population size, the so-called ‘large p, small n’ problem, markedly impact the resolution of recombination so that only a minority of markers can be actually ordered (Ronin et al., 2010). On the other hand, high-density genetic maps usually result in many markers clustering at the same position (co-segregating markers) on the linkage map; e.g., (Liu et al., 2013; Iehisa et al., 2014; Talukder et al., 2014; Zhou et al., 2015; Di Pierro et al., 2016; Liu Z. et al., 2016; Ren et al., 2016; Tyrka et al., 2016). In spite of the availability of several papers on genetic mapping, specific studies related to the impact of high throughput SNP data on genetic maps have not yet been conducted. It is therefore timely to consider how the increasing number of markers can impact genetic map features in the era of high throughput sequencing technologies.

Machine learning (ML) is the study of data-driven, computational methods for making inferences and predictions (Breiman, 2001b) and may be seen as the intersection of Computer Science and Statistics (Cunningham, 1995). ML methods have been applied in diverse fields, including face recognition, speech processing (e.g., Google voice, Apple’s Siri), prediction of consumers preferences (e.g., Netflix movie recommender system), text mining (Witten and Eibe, 2005), bioinformatics [e.g., drug design and genome annotation (Yip et al., 2013) or transcription networks (Li et al., 2006)], cell biology (Sommer and Gerlich, 2013), medical diagnosis (Kukar and Groselj, 2005) and disease tissue classification in medicine (Guyon et al., 2002; Zacharaki et al., 2009). Due to their high generalization capabilities and distribution-free properties, ML algorithms are presented as a valuable alternative to traditional statistical techniques (Maenhout et al., 2010). Moreover, ML algorithms can deal with heterogeneity of the data, redundancy and presence of interactions and non-linearity (Ornella et al., 2012).

In animal and crop breeding, ML algorithms have been widely used in the framework of genomic selection (GS), e.g., (Bernardo and Yu, 2007; Goddard and Hayes, 2007; Gianola and van Kaam, 2008; Gonzales-Recio et al., 2008; Jannink et al., 2010; Heslot et al., 2012; Grinberg et al., 2016). GS (Meuwissen et al., 2001) uses all available DNA marker information across the genome to estimate genetic values (Bernardo, 2008; Jannink et al., 2010) for improved selection of quantitative trait. GS uses a training population (set of individuals having genotypic and phenotypic data) to develop a model to predict genomic estimated breeding values (GEBVs) of non-phenotyped individuals. There is an increasing interest in ML for use in other aspects of crop breeding, including high throughput phenotyping (Mahlein, 2015; Singh et al., 2016; Wahabzada et al., 2016) and determining the most important features that contribute to agronomic traits of interest (Ornella et al., 2012; Shaik and Ramakrishna, 2014; Shekoofa et al., 2014).

The objective of our study is to investigate the effects of co-segregating markers on high-density genetic map length and marker order using empirical data from durum and bread wheat. Ultimately, we aim to predict the inflation factor (IF) of the linkage maps, using ML algorithms.

## Materials and Methods

### Plant Material

Two doubled haploid mapping populations described elsewhere were used in this study: the durum wheat Mohawk × Cocorit (Maccaferri et al., 2014) and the bread wheat Norstar × Cappelle Desprez (Fowler et al., 2016). The Mohawk × Cocorit and Nortar × Cappelle Desprez populations consisted of 177 and 256 lines, respectively.

### Genotyping

As described in earlier publications (Maccaferri et al., 2014; Fowler et al., 2016), DNA of the mapping populations was extracted from young leaves using the DNeasy 96 Plant Kit (QIAGEN Science, Germantown, MD, United States). DNA was quantified using NanoDrop ND-1000 UV-vis spectrophotometer (Thermo Fisher Scientific Inc., Madison, WI, United States). Genotyping was performed at the Crop Development Centre, University of Saskatchewan using the Illumina Infinium wheat 90K iSelect assay (Illumina Inc., San Diego, CA, United States) as reported previously (Wang et al., 2014). The raw intensity data were processed with the GenomeStudio v2011.1 software (Illumina). Genotypic data were curated to correct for scoring errors, filter out monomorphic and highly distorted markers according to the expected 1:1 ratio for DH populations using chi-square (χ^2^) test as implemented in the MapDisto software (Lorieux, 2012).

### Mapping Procedure

Our approach consisted of two phases with the following steps:

#### Phase I

– For each population, all curated SNP data was used to build linkage maps using the MSTMap software (Wu et al., 2008) with a stringent cut off *p*-value of 1E^-10^ and a maximum distance between markers of 15.0 cM for clustering SNPs into linkage groups (LGs). Double recombinants were corrected using the functions ‘Show double recombinants,’ ‘Show error candidates’ and ‘Replace error candidates by flanking genotype’ as implemented in the MapDisto software (Lorieux, 2012). The LGs were assigned to chromosomes based on existing high-density SNP maps (Cavanagh et al., 2013; Maccaferri et al., 2014; Wang et al., 2014).
– For each LG, a skeleton map was built by keeping only one of the most informative (highest polymorphism information content, lowest number of missing data) markers randomly selected per cluster (group of markers located at the same position).
– Then, using an in-house Ruby script, we built as many maps (hereafter referred to sequential maps) as there were co-segregating markers on each LG (see step 1) by adding one marker at a time (one after another), selected randomly from the list of co-segregating markers.

#### Phase II

– Because LGs had different sizes and the number of co-segregating markers varied among them, we computed the proportions of co-segregating markers relative to the total number of markers on each LG.
– Eight levels of proportion, ranging from 10 to 80% were sampled for all LGs having ≥80% of co-segregating markers.
– Each proportion level had 50 replicates. For example, for LG 1A we randomly selected 10% of co-segregating markers 50 times to build 50 ‘sequential’ maps. Then, we repeated the same process for 20, 30, 40, 50, 60, 70, and 80% of co-segregating markers. However, LGs 2A, 4A and 5A in Mohawk × Cocorit and 1D, 4D and 7D in Norstar × Cappelle Desprez had less than 80% of co-segregating markers and only six proportion levels (10–60%) with 20 replicates were used.
– The length of these sequential maps and markers order were compared to those of the skeleton map.
– Finally, for each sequential map the IF was estimated as:
  IF = ((*L*_seq_ – *L*_sket_)/ *L*_sket_) ∗ 100,
  *L*_seq_ and *L*_sket_ being the length of the sequential map and the skeleton map, respectively.

### Prediction

Seven ML algorithms implemented in the Caret R package (Kuhn et al., 2012) were used to predict the inflation of the map lengths relative to the proportion of co-segregating markers:

– Linear regression model (LR): LR was developed in the field of statistics, but has been borrowed by ML. The LR algorithm is a family of model-based learning approaches that assume a linear relationship between the input variables (x) and the single output variable (y). The LR equation is built and trained, using different techniques, the most common of which is called Ordinary Least Squares (OLS). The OLS is a method for estimating the unknown parameters in a LR while minimizing the sum of the squares of the differences between the observed responses (values of the variable being predicted) in the given dataset and those predicted by a linear function of a set of explanatory variables.
– Generalized linear model (GLM): The GLM provides flexible generalization of ordinary linear regression for response variables with error distribution models other than a Gaussian (normal) distribution. GLM unifies various other statistical models, including binomial, gamma, Poisson and logistic regression. Each serves a different purpose, and depending on distribution and link function, GLM can be used for prediction or classification.
– Polynomial regression with degree 2 (POLY2) and 3 (POLY3): Polynomial regression is a form of linear regression in which the relationship between the input variables (x) and the output variable (y) is modeled as a polynomial. Although polynomial regression fits a non-linear model to the data, it is considered as a special case of multiple linear regression since it is linear in the regression coefficients. We only tried quadratic (POLY2) and cubic (POLY3) models to avoid overfitting.
– K-nearest neighbors (KNN): The KNN algorithm is an instance-based learning where new data are classified based on stored, labeled instances. The rationale behind the KNN algorithm is learning by analogy. The distance between the stored data and the new instance is calculated using similarity measures such as the Euclidean distance, cosine similarity or the Manhattan distance. The similarity value is used to perform predictive modeling for classification or regression. In both cases, the input consists of the k closest training examples in the feature space. For classification, the output is a class membership while for regression, it is the property value for the object. This value is the average of the values of its k nearest neighbors.
– Support vector machine (SVM) (Vapnik, 1995): SVM uses a non-linear mapping function to map samples from the predictor space to a high-dimensional feature space and perform linear regression in the latter space (Witten and Frank, 2005).
– Classification and regression trees (CART) (Breiman et al., 1984): CART is a decision tree algorithm for both classification and regression. It is a recursive algorithm, which partitions the training data set by doing binary splits. In their simplest form, decision tree algorithms are hierarchical if-else statements that can be applied to predict a result based upon data. The if-else statements are chosen to maximize a notion of information gain and reduce the variability in the underlying (two) children nodes. In contrast with general tree-based methods that may allow multiple child nodes, CART always creates a binary tree. A large tree is first generated, then pruned to a size that has the lowest cross-validation estimate of error (Loh, 2014).
– Random forest (RF) (Breiman, 2001a): RF is an ensemble algorithm based on randomized regression trees. In RF, each tree is built from a sample drawn with replacement (i.e., a bootstrap sample) from the training set. Each tree individually predicts the target response and the ‘forest’ (i.e., the ensembles of ‘trees’) predicts the target response as an average of individual tree predictions.

To evaluate the map expansion, only maps generated using different proportion levels (10–80%) of co-segregating markers were used, 4800 and 7580 maps for Mohawk × Cocorit and Norstar × Cappelle Desprez, respectively. Two types of partition designs were used to build the prediction models. In the first partition design, the whole set of sequential maps for each population was split into training and test sets containing 80 and 20% of the maps, respectively. The second partition design was a 10-fold cross-validation scheme with 5 replicates (Kohavi, 1995). The data was divided into 10 sets to which maps were assigned randomly so that all sets consisted of equal number of maps. One subset (testing set) was omitted to test the predictive ability of the model, whereas the other nine subsets were used as training samples (training set) to estimate model parameters. During cross-validation runs, each of the 10 subsets served as a testing set in one round, with missing values.

The models were fitted using the training sample, and the fitted models were used to predict outcomes in the test set. The goodness-of-fit of the models was evaluated using the root mean square error (RSME). The prediction accuracy was estimated as a Pearson correlation between the predicted and the observed map length in the test set.

## Results

### Description of the Linkage Maps

A total 24,473 linkage maps were built for this study: 8,203 maps for Mohawk × Cocorit and 16,270 maps for Norstar × Cappelle Desprez populations.

The features of Mohawk × Cocorit and Norstar × Cappelle Desprez maps that were built in step 1 of phase I are presented in **Table 1** and **Table 2**, respectively. For Mohawk × Cocorit, the map using the whole curated data set consisted of 3,999 SNPs spanning 2421.1 cM. Markers were distributed on the 14 chromosomes of the durum wheat genome. The number of markers per chromosome varied from 76 (chromosome 4A) to 529 (chromosome 6B). In total, 85% (3,389/3,999) of the markers co-segregated across the genome. The proportion of co-segregating markers of genome A was lower than that of genome B (81 vs. 87%).

**Table 1:** Features of the Mohawk × Cocorit linkage map.

	Full map		Skeleton map		Co-segregating markers	
Chromosomes	Markers	Map size (cM)	Markers	Map size (cM)	Number	Proportion (%)
1A	348	154.4	58	137.3	290	83
1B	277	205.9	46	197.2	231	83
2A	90	183.6	28	148.7	62	69
2B	334	161.1	51	145.0	283	85
3A	269	90.3	27	82.2	242	90
3B	323	231.8	55	180.3	268	83
4A	76	141.7	24	128.9	52	68
4B	340	156.9	40	119.9	300	88
5A	91	71.7	36	63.7	55	60
5B	323	207.8	36	178.4	287	89
6A	300	200.5	63	165.7	237	79
6B	529	215.7	53	188.9	486	92
7A	330	247.2	64	192.6	276	84
7B	369	152.5	49	123.2	320	87
Genome A	1504	1089.4	300	919.1	1214	81
Genome B	2495	1331.7	330	1132.9	2175	87
Total	3999	2421.1	630	2052.0	3389	85

**Table 2:** Features of the Norstar × Cappelle Desprez linkage map.

	Full map		Skeleton map		Co-segregating markers	
Chr	Markers	Map size (cM)	Markers	Map size (cM)	Number	Proportion (%)
1A	909	107.1	85	90.6	824	91
1B	673	235.9	122	217.9	551	82
1D	102	110.8	31	103.0	71	70
2A	483	228.5	95	212.9	388	80
2B	864	230.7	96	216.2	768	89
2D	498	198.8	42	185.9	456	92
3A	593	246.2	94	219.3	499	84
3B	681	253	129	226.1	552	81
3D	76	18.7	6	17.8	70	92
4A	398	188.6	70	179.0	328	82
4B	424	130.1	68	127.9	356	84
4D	29	15.3	8	14.4	21	72
5A	636	281.4	117	278.9	519	82
5B	1049	225.9	126	211.6	923	88
5D	107	27.9	13	19.8	94	88
6A	437	170.6	66	156.7	371	85
6B	937	185.0	101	170.3	836	89
6D	103	47.5	15	15.8	91	88
7A	641	216.9	114	200.7	527	82
7B	471	144.6	70	133.6	401	85
7D	43	72.1	20	71.6	23	53
Genome A	4097	1439.3	641	1338.1	3456	84
Genome B	5099	1405.2	712	1303.6	4387	86
Genome D	958	491.1	135	428.3	826	86
Total	10154	3335.6	1488	3070.0	8669	85

For Norstar × Cappelle Desprez, 10,154 markers spanning 3335.6 cM were mapped on the 21 chromosomes of the bread wheat genome. The genome-wide proportion of co-segregating markers was 85% (8,669/10,154), ranging from 53 (chromosome 7D) to 92% (chromosomes 2D and 3D). Genome A displayed 84% of co-segregating markers while genomes B and D showed 86% of co-segregating markers.

Markers order analysis revealed a very high collinearity between sequential maps and the skeleton map for all chromosomes in both Mohawk × Cocorit and Norstar × Cappelle Desprez (**Table 3**). The average Spearman correlation coefficient ranged from 0.94 to 0.99 and 0.97 to 0.99 for Mohawk × Cocorit and Norstar × Cappelle Desprez, respectively.

**Table 3:** Spearman correlation coefficient of markers order between sequential maps and skeleton map in Mohawk × Cocorit and Norstar × Cappelle Desprez.

Chromosomes	Mohawk × Cocorit	Norstar × Cappelle Desprez
1A	0.99	0.99
1B	0.97	0.99
1D		0.99
2A	0.94	0.99
2B	0.99	0.99
2D		0.98
3A	0.99	0.99
3B	0.95	0.99
3D		0.97
4A	0.97	0.99
4B	0.96	0.99
4D		0.98
5A	0.96	0.99
5B	0.99	0.99
5D		0.99
6A	0.97	0.99
6B	0.98	0.99
6D		0.97
7A	0.99	0.99
7B	0.99	0.99
7D		0.99

### Maps Expansion

**FIGURE 1:**
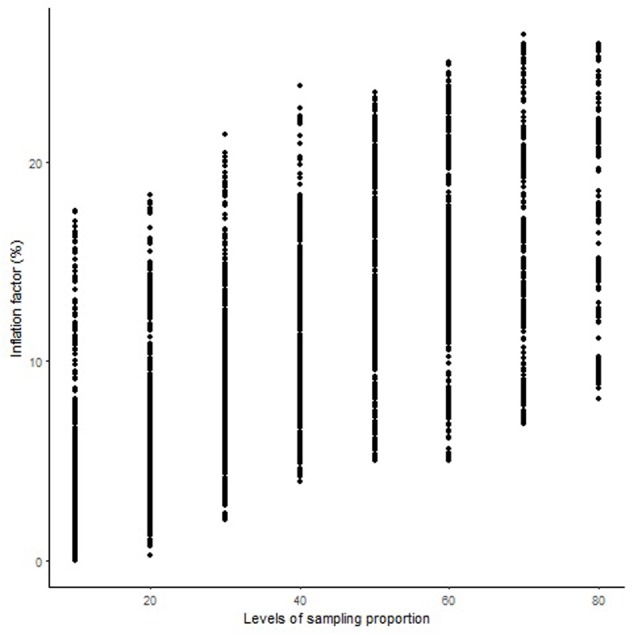
Genome-wide pattern of map length inflation factor in the Mohawk × Cocorit population.

**FIGURE 2:**
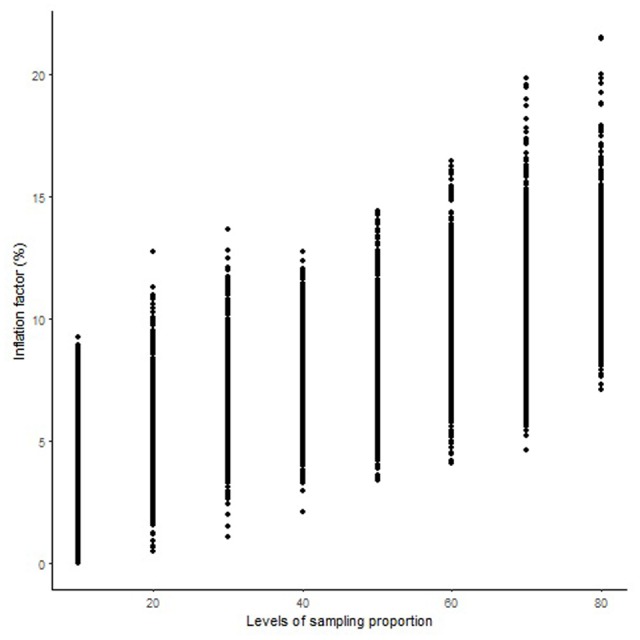
Genome-wide pattern of map length inflation factor in the Norstar × Cappelle Desprez population.

The length of the sequential maps expanded in proportion to the co-segregating markers for both Mohawk × Cocorit (**Figure 1**) and Norstar × Cappelle Desprez (**Figure 2**). For a given proportion of co-segregating markers genome-wide, there was a relatively wide range variation of the IF, e.g., with 80% co-segregating markers IF ranged from 8 to 25% and 7 to 21% in Mohawk × Cocorit and Norstar × Cappelle Desprez, respectively.

**FIGURE 3:**
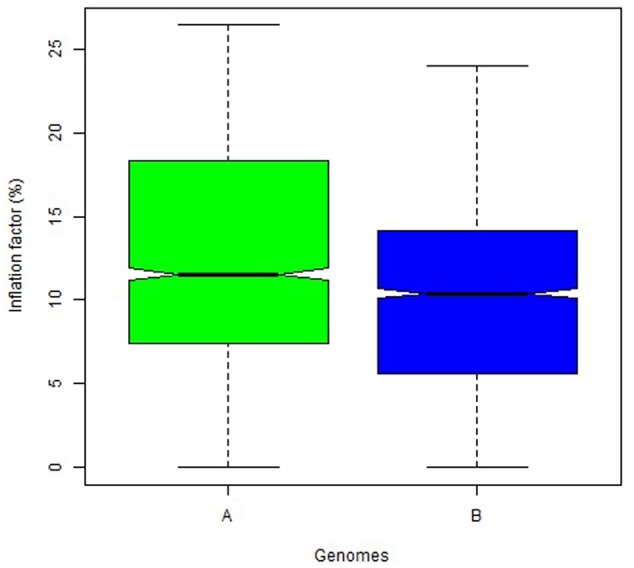
Boxplot of map length inflation factor per genome in the Mohawk × Cocorit population.

**FIGURE 4:**
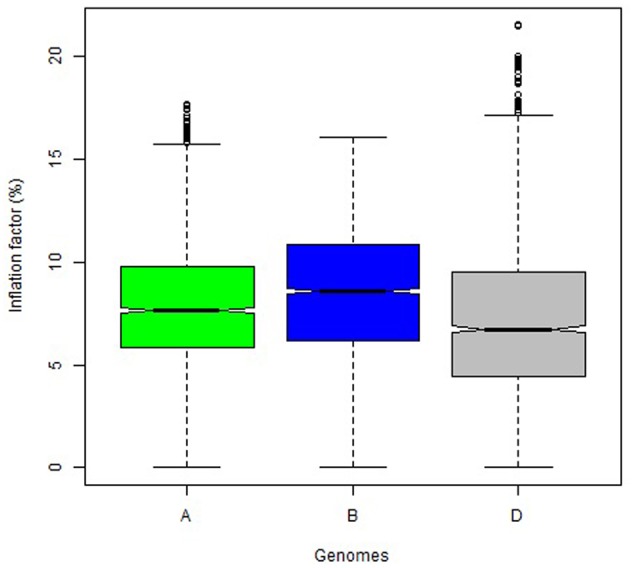
Boxplot of map length inflation factor per genome in the Norstar × Cappelle Desprez population.

**FIGURE 5:**
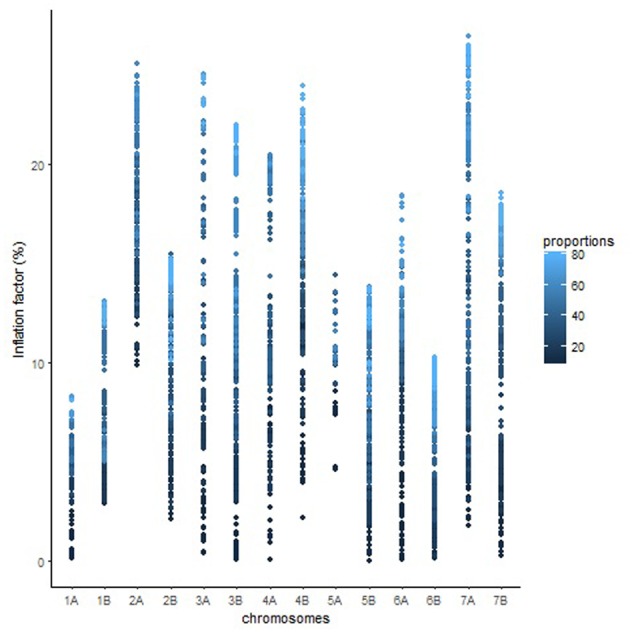
Pattern of inflation factor for chromosomes and the proportions of co-segregating markers in the Mohawk × Cocorit population.

**FIGURE 6:**
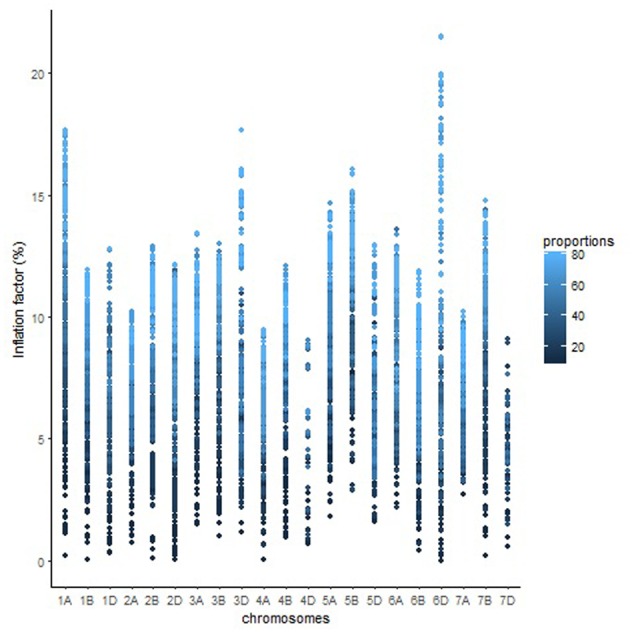
Pattern of inflation factor for chromosomes and the proportions of co-segregating markers in the Norstar × Cappelle Desprez population.

The overall variation in IF was similar among genomes in Mohawk × Cocorit (**Figure 3**) and Norstar × Cappelle Desprez (**Figure 4**). However, a few outliers were observed in genomes A and D in Norstar × Cappelle Desprez. Despite of the relatively wide variation of IF within chromosome, the higher proportion of co-segregating markers the larger the IF for both Mohawk × Cocorit (**Figure 5**) and Norstar × Cappelle Desprez (**Figure 6**). For example in Mohawk × Cocorit, the average IF on chromosome 3B for 10, 50, and 80% of co-segregating markers was 2.1, 12.7, and 21.6%, respectively. Similarly, in Norstar × Cappelle Desprez the average IF on 3B for 10, 50, and 80% of co-segregating markers was 3.6, 9.8, and 11.8%, respectively.

### Prediction of Map Expansion

The prediction accuracies of the models are shown in **Table 4**. All of the models resulted in similar performance (RMSE, accuracy) in both Mohawk × Cocorit and Norstar × Cappelle Desprez populations. The IF of the linkage maps was predicted with an accuracy of 0.7 in both populations. However, the RMSE was lower in Norstar × Cappelle Desprez compared to Mohawk × Cocorit, 2.2 vs. 4.6. The map length inflated relative to the proportion of co-segregating markers: the more co-segregating markers the larger the map expansion (**Table 5**). For example in Mohawk × Cocorit, with 10 and 80% co-segregating markers the length of the map inflated by 4.5 and 16.6%, respectively. Similarly, the map of Norstar × Cappelle Desprez expanded by 3.8 and 11.7% with 10 and 80% co-segregating markers.

**Table 4:** Prediction accuracy of different models in the Mohawk × Cocorit and Norstar × Cappelle Desprez populations.

Populations	Models^1^	RMSE^2^	Accuracy
Mohawk × Cocorit	LR	4.631	0.654
	GLM	4.631	0.654
	KNN	4.577	0.664
	POLY2	4.584	0.664
	POLY3	4.578	0.664
	SVM	4.632	0.661
	CART	4.694	0.638
	RF	4.577	0.664
Norstar × Cappelle Desprez	LR	2.234	0.737
	GLM	2.234	0.737
	KNN	2.225	0.742
	POLY2	2.234	0.737
	POLY3	2.229	0.739
	SVM	2.227	0.743
	CART	2.389	0.667
	RF	2.225	0.742

*^1^Models: LR, linear regression model; GLM, generalized linear model; KNN, K-nearest neighbors; POLY2, quadratic regression; POLY3, cubic regression; SVM, Support vector machine; CART, Classification and regression trees; RF, Random forest. ^2^RMSE: Root mean square error.*

**Table 5:** Map inflation factor (mean ± standard deviation) relative to the proportion of co-segregating markers in the Mohawk × Cocorit and Norstar × Cappelle Desprez populations.

	Mohawk × Cocorit		Norstar × Cappelle Desprez	
Co-segregating markers (%)	Number of maps	Inflation factor (%)	Number of maps	Inflation factor (%)
10	700	4.48 (±3.63)	990	3.77 (±1.99)
20	700	6.85 (±3.94)	990	5.43 (±2.02)
30	700	9.34 (±3.79)	990	6.71 (±2.02)
40	700	11.11 (±4.08)	990	7.39 (±1.95)
50	700	13.78 (±4.95)	990	8.24 (±2.34)
60	700	14.86 (±5.06)	970	9.35 (±2.46)
70	650	16.59 (±5.52)	970	10.85 (±2.65)
80	650	16.62 (±5.58)	950	11.70 (±2.28)

## Discussion

### Linkage Mapping

All of the linkage maps were constructed using MSTMap software (Wu et al., 2008) due to its good performance compared to other available tools, especially in the speed and accuracy of map construction (Cheema and Dicks, 2009). In this study, we built a total of 24,473 linkage maps by taking advantage of the fast algorithm of MSTMap combined with an in-house Ruby script that generated the appropriate data sets, parameter files and ran MSTMap in batch mode. A wide range of algorithms and software for constructing genetic maps are available (see Cheema and Dicks, 2009; Ott et al., 2015 for a review). In particular, many algorithms have been designed for high-density maps (van Os et al., 2005a,b; Rastas et al., 2013; Liu et al., 2014). Algorithms usually try to solve specific mapping problems such as correcting genotyping errors (van Os et al., 2005b; Liu et al., 2014), producing accurate marker order in a relatively limited time (van Os et al., 2005a), handling populations with highly heterozygous loci (Margarido et al., 2007; Tong et al., 2010) or detecting and removing pseudo-linkages (Ronin et al., 2010).

No single software harbors all the desirable features (e.g., ultra-fast, accurate in makers order, no map inflation, scalable) that one could expect for assembling a high quality high-density map in a relatively short time. Therefore, different combinations of software have been used to build high-density genetic maps (e.g., Liu et al., 2014, 2015; Fowler et al., 2016; Kumar et al., 2016; Perez-Lara et al., 2016). Fowler et al. (2016) and Perez-Lara et al. (2016) used MSTMap + MapDisto (Lorieux, 2012) while Liu et al. (2014) used AntMap (Iwata and Ninomiya, 2006) + MapDisto. In contrast, Kumar et al. (2016) combined the features of MapMaker (Lander and Botstein, 1989) and CarthaGene (de Givry et al., 2005) while Liu et al. (2015) built their map using JoinMap (Stam, 1993) + MSTMap. Several practical strategies have also been used to tackle the difficulties in constructing a high-density linkage map, including bin strategy (Sun et al., 2007; Amores et al., 2011; Ganal et al., 2011; Chen Z. et al., 2014; Han et al., 2016; Zhou et al., 2016). Bin strategy reduces computational costs as well as the impact of genotyping errors, but at the cost of incomplete utilization of genotyping data and recombination information reducing the application value of high-density linkage map (Liu et al., 2014). Another approach, termed ‘selective mapping,’ suggests first building a framework map with limited number of markers and samples of individuals bearing complementary recombination breakpoints, then adding the remaining markers (Vision et al., 2000). Similarly, Ronin et al. (2010) recommended use of ‘delegate’ markers to build a reliable skeleton map and eventually remove markers that create local instability. It’s well known that different mapping strategies may result in different maps (Ronin et al., 2010). However, to avoid any potential technical bias in our study, all of the maps were constructed using the same software and algorithms.

### Markers Order

A strong collinearity in markers order (*r* ranging for 0.94 to 0.99) was observed between the sequential maps and the skeleton map for all chromosomes in both Mohawk × Cocorit and Norstar × Capelle Desprez populations suggesting that co-segregating markers had little effect on markers order. The ordering of markers within LGs is considered a special case of the classical traveling salesman problem (Doerge, 1996; Liu, 1998; Mester et al., 2003; Tan and Fu, 2006). The problem consists in choosing the best order among (1/2)^∗^m! possible orders (m being the number of markers). When m gets larger, the number of orders is unwieldy. For example, when *m* = 100, the total number of possible orders = 4.6 × 10^157^, which is not feasible with the currently available computational power. Algorithms to obtain approximate optimal solutions are the only practical approach for large-scale linkage mapping (Liu, 1998). Thus, some small local discrepancies in marker order might occur when comparing maps. However, most of the linkage mapping algorithms find reasonably good markers order (see Wu et al., 2008 for a review).

### Map Expansion

“Map expansion is the phenomenon that genetic maps including a large number of genes are longer than the corresponding actual genetic distance between the genes involved” (Sybenga, 1996). Discrepancies between genetic maps and cytological maps have raised some concerns about map expansion (Hall et al., 1997a,b) in many species, including mice (Taylor, 1978), maize (Burr et al., 1988; Burr and Burr, 1991; Anderson et al., 2003), tomato (Paran et al., 1995) and potato (Tanksley et al., 1992).

Many sources of map expansion have been reported, including genotyping errors and missing values (Lincoln and Lander, 1992; Sobel et al., 2002; Hackett and Broadfoot, 2003; Pompanon et al., 2005; Cartwright et al., 2007; Avni et al., 2014; Ronin et al., 2014), number and type of markers (Lee et al., 2015; Bai et al., 2016), tight double recombinant events, and segregation distortion (Sybenga, 1996) and mapping software (Sybenga, 1996; Hackett and Broadfoot, 2003; Falque, 2005; Rastas et al., 2016). Other factors, including an excess of heterozygosity (Knox and Ellis, 2002; Truong et al., 2014) and the population type and size (Ferreira et al., 2006; Lee et al., 2015) have also been reported to inflate the length of linkage maps.

Nonetheless, only the correction of genotyping errors and a reduction in missing values have led to substantial improvement of algorithms for the construction of high-density linkage maps (Lincoln and Lander, 1992; Stam, 1993; Douglas et al., 2000; van Os et al., 2005b; Cartwright et al., 2007; Ronin et al., 2010, 2014; Lorieux, 2012). Genotyping errors can unlink markers that would be identical (absolutely linked) in the ideal situation with no errors. When the number of markers and the error rate increase, it becomes more challenging to build a reliable map (Ronin et al., 2010). As marker density increases, undetected scoring errors rate of only 1% can lead to incorrect markers order and map expansion (Buetow, 1991). More precisely, it was reported that every 1% error rate in a marker data inflates the map length by 2 cM (Cartwright et al., 2007). While missing values leads to a poor estimate of the true recombinations that have occurred along the chromosome. A common practice to deal with missing data is imputation (Zhao et al., 2008; Marchini and Howie, 2010; Daetwyler et al., 2011; Schwender, 2012). However, missing values have a limited negative impact on the accuracy of the final map, compared to genotyping errors (Hackett and Broadfoot, 2003; Wu et al., 2008), provided that the number of missing values remains relatively low. For this reason, some authors prefer keeping ambiguous genotypes as missing data rather than inferring the putative alleles (Wu et al., 2008). Although some authors analyzed data having up to 80% missing values (e.g., Edae et al., 2016), we kept this rate relatively low to reduce their impact on the map expansion (Hackett and Broadfoot, 2003; Wu et al., 2008). Only SNP with less than 10% missing data were used for our analyses. Therefore, missing data had limited contribution to the map expansion we observed.

The effect of co-segregating markers on linkage maps has received less attention. However, our study clearly showed that an excess of co-segregating markers leads to map expansion. The more co-segregating markers, the larger the map expansion. Using ML approaches, we were able to predict with an accuracy of 0.7 the map expansion relative to the proportion of co-segregating markers. Although we used both linear and non-linear methods, all of the ML algorithms gave similar results supporting evidence of a linear relationship between map expansion and the number of co-segregating markers. The proportion of co-segregating markers ranged from 60 to 92% in Mohawk × Cocorit (**Table 1**) and 53 to 92% in Norstar × Cappelle Desprez (**Table 2**), with an average of 85% in both populations. This relatively high proportion of co-segregating markers is not exceptional since the ‘large p, small n’ problem, derived from high-throughput data has not yet been resolved by any mapping algorithm. Intuitively, all of the high-density genetic maps in the literature contain a high proportion of co-segregating markers, regardless of the species. Because this metric is not reported for published genetic maps, we computed it for some species where map data are available online. For example, the genome-wide proportions of co-segregating markers were 75% (14023/18601) in a wheat MAGIC map (Gardner et al., 2016), 65% (8408/12998) in barley (Zhou et al., 2015), 57% (2948/5138) in sunflower (Talukder et al., 2014) and 70% (6426/9164) in *Brassica napus* (Liu et al., 2013). For pearl millet, it was reported that only 314 out of 2,156 SNPs showed unique map position, giving 85% co-segregating markers (Moumouni et al., 2015).

To deal with map expansion, a common practice is to remove the double recombinants. However, the method of removing erroneous double recombinants could lead to irrelevant distances among markers (Ronin et al., 2010). As an example, Ronin et al. (2010) applied that method to chromosome 1B of a recombinant inbred line population of wheat and produced a map of 104 cM, compared to the published map that spanned 432 cM. The relatively small length of the map was attributed to an artifact introduced during the merging of different marker data sources, some of which contained high frequencies of missing data and inappropriate “error correction.” Another approach is to adjust the length of the map based on the breeding scheme, in particular for RIL and IRIL (Winkler et al., 2003). However, some studies have shown that the IF derived from this method tends to be underestimated with low marker density (Teuscher et al., 2005; Liu et al., 2015).

We estimated the IF of each LG with respect to the length of its skeleton map. Because only a few markers can reliably be ordered in a context of high-density linkage mapping where the number of markers exceed by far the size of the population (Ronin et al., 2010), many authors suggested first building a skeleton map with ‘delegate’ markers, before adding the remaining markers, e.g., (van Os et al., 2006; Peleg et al., 2008; Wu et al., 2008; Ronin et al., 2010; Seetan et al., 2013; Reddy et al., 2014; Strnadová et al., 2014; Mester et al., 2015). In many studies, only the skeleton map was used to perform analyses such as QTL detection (Chutimanitsakun et al., 2011; Vengadessan et al., 2013; Chen Z. et al., 2014; Liu J. et al., 2016) or as a reference to calculate the genetic distances between markers (Ren et al., 2012; Moumouni et al., 2015). Thus, skeleton maps appear to be the backbone of high-density genetic maps.

Machine learning algorithms are becoming more accepted in crop breeding and are presented as a worthwhile surrogate to traditional statistical methods (Maenhout et al., 2010). The predictive ability of ML algorithms has proven superior to classical statistics methods in many studies (Drummond et al., 2003; Gonzalez-Sanchez et al., 2014). In particular, ML algorithms have been successfully applied to crop yield prediction (see for Mishra et al., 2016 a review), including wheat (Jeong et al., 2016; Pantazi et al., 2016), maize (Liu et al., 2001; Marinkovic et al., 2009; Jeong et al., 2016), potato (Al-Hamed and Wahby, 2016; Jeong et al., 2016) and cotton (Zhang et al., 2008). Due to their high predictive performance and high generalization capabilities, ML algorithms are becoming a valuable tool for data mining.

Because of the continued increase in the size of high throughput SNP-chips, the disparity between the high number of markers and the relatively small population size is more likely to result in poor resolution maps (Ronin et al., 2010). Intuitively, the proportion of co-segregating markers in high-density maps will continue to increase, making map expansion unavoidable. Therefore, there is a need for improved linkage mapping algorithms to efficiently analyze the high-throughput data generated by new sequencing technologies. In particular, developers should build algorithms capable of computing accurately recombination frequencies and genetic distances in a context of high-density linkage mapping.

## Conclusion

Our study clearly showed that excess of co-segregating markers can lead to map expansion with little effect on markers order. Using various ML algorithms, we were able to predict with an accuracy of 0.7 map expansion relative to the proportion of co-segregating markers. Because co-segregating markers are inevitable in high-density linkage maps, it becomes necessary to improve linkage mapping algorithms for efficient analysis of high-throughput data. In the meantime, a practical strategy could be to estimate the IF related to the proportion of co-segregating markers and then scale the length of the map accordingly.

## Author Contributions

AN’D set up the experimental design, analyzed the data, and wrote the initial manuscript. JH edited the manuscript. DF and KA created the mapping populations and edited the manuscript. CP provided all resources including funding, designed the experiment and edited the manuscript.

## Conflict of Interest Statement

The authors declare that the research was conducted in the absence of any commercial or financial relationships that could be construed as a potential conflict of interest.
